# Identifying Stress Transcription Factors Using Gene Expression and TF-Gene Association Data

**DOI:** 10.4137/bbi.s292

**Published:** 2009-11-24

**Authors:** Wei-Sheng Wu, Bor-Sen Chen

**Affiliations:** Lab of Control and Systems Biology, Department of Electrical Engineering, National Tsing Hua University, Hsinchu, 300, Taiwan

**Keywords:** stress, transcription factor, gene expression data and TF-gene association data

## Abstract

Unicellular organisms such as yeasts have evolved to survive environmental stresses by rapidly reorganizing the genomic expression program to meet the challenges of harsh environments. The complex adaptation mechanisms to stress remain to be elucidated. In this study, we developed Stress Transcription Factor Identification Algorithm (STFIA), which integrates gene expression and TF-gene association data to identify the stress transcription factors (TFs) of six kinds of stresses. We identified some general stress TFs that are in response to various stresses, and some specific stress TFs that are in response to one specific stress. The biological significance of our findings is validated by the literature. We found that a small number of TFs may be sufficient to control a wide variety of expression patterns in yeast under different stresses. Two implications can be inferred from this observation. First, the adaptation mechanisms to different stresses may have a bow-tie structure. Second, there may exist extensive regulatory cross-talk among different stress responses. In conclusion, this study proposes a network of the regulators of stress responses and their mechanism of action.

## Introduction

Single-celled organisms such as yeasts regularly face variable and often harsh external environments which threaten its survival or at least prevent it from performing optimally. Environmental changes may be of a physical or chemical nature: temperature, oxidation, osmolarity, acidity, nutrient availability, etc (Hohmann and Mager, 2003). Yeasts have evolved to survive environmental stresses by rapidly responding to changes in environmental conditions. The adaptation mechanisms to stress are highly complex. One aspect of this cellular adaptation is the reorganization of genomic expression. The genomic expression program required for maintenance of the optimal cell physiology in one environment may be far from optimal in a different environment. Thus, when environmental conditions change abruptly, the cell rapidly adjusts its genomic expression program to adapt to the new conditions (Gasch et al. 2000).

The reprogramming of genomic expression can be unveiled using genome-wide DNA microarrays, which measure the relative transcript levels of essentially every gene in the yeast genome at any given moment, providing a snapshot of the genomic expression program (Gasch and Werner-Washburne, 2002). Exploring the dynamic nature of the yeast genome through time-course experiments can illuminate the yeast stress response. For example, Gasch et al. (2000) and Causton et al. (2001) used genome-wide expression analysis to explore how gene expression in yeast is remodeled over time as cells respond to heat shock, oxidative shock, osmotic shock, acidic stress, nitrogen depletion, amino acid starvation, etc. They discovered that more than half of the genome is involved in responding to at least one of the investigated environmental changes. A set of genes (~10% of yeast genes), termed as the environmental stress response (ESR) genes or common environmental response (CER) genes, showed a similar drastic response to almost all of these environmental changes. Other gene expression responses appeared to be specific to particular environmental conditions. Characterizing environmentally triggered gene expression changes provides insights into when each gene is expressed (Gasch and Werner-Washburne, 2002). However, the complete network of the regulators of stress responses and the details of their actions remain to be elucidated (Gasch et al. 2000).

In this study, we try to identify the stress TFs of six kinds of stresses. We used two kinds of data sets. First, genome-wide gene expression time profiles under various stress conditions such as heat shock, oxidative shock, osmotic shock, acidic stress, nitrogen depletion, and amino acid starvation are from Gasch et al. (2000) and Causton et al. (2001). Under each stress condition, samples are collected at multiple time points for all genes in the yeast genome. Each Cy5-labeled sample was compared with a Cy3-labeled reference pool, consisting of an equal mass of all of the RNA samples. Then the data were mathematically “zero transformed” for visualization by dividing the expression ratios for each gene measured on a given array by the corresponding ratios measured for the unshocked, time-zero cells. Therefore, the ratios represent the expression level at each time point relative to the expression level in the unshocked, time-zero sample. Second, genome-wide regulatory targets of a TF are retrieved from YEASTRACT database (Teixeira et al. 2006b). The regulatory associations between genes and TFs are reported if they are supported by experimental results from the literature or genome-scale studies such as motif data (TF-binding sites), mutant data (genome-wide gene expression changes in mutant strains defective in TFs), and ChIP-chip data (genome-wide binding targets of TFs). Therefore, it is a comprehensive data set to include all possible TF-gene associations under various kinds of conditions. Total of 147 TFs, which are collected in YEASTRACT database, are used in this study. We developed Stress Transcription Factor Identification Algorithm (STFIA), which integrates these two kinds of high throughput genome-scale data (gene expression and TF-gene association data) to identify plausible stress TFs that regulate the target genes to confer stress protection.

## Methods

### Stress transcription factor identification Algorithm (STFIA)

**Step 1:** (identification of the stress-responsive genes)

STFIA finds out the genes in the yeast genome that respond to a specific stress. A gene is said to be in response to a specific stress if more than *T* points of its expression time profile measured under that specific stress are induced or repressed by more than *F* fold compared to that of the unstressed condition. (*T* and *F* are two parameters to be specified.)

**Step 2:** (identification of the stress TFs)

For each of the 147 TFs, STFIA determines whether the TF under study is involved in responding to a specific stress. The rationale is that a TF is involved in responding to a specific stress if a statistically significant portion of its regulatory targets is in response to that specific stress. The hypergeometric distribution is used to test the statistical significance (Mendenhall and Sincich, 1995). The *p*-value computed from the hypergeometric model is then adjusted by Bonferroni correction to represent the true alpha level in the multiple hypothesis testing (Men-denhall and Sincich, 1995). A TF is said to be involved in responding to a specific stress if the adjusted *p*-value *p_adjusted_* ≤ *p_threshold_* where *p_threshold_* is a parameter to be specified.

For the illustrative purpose, let us take Hsf1, a well-known heat shock TF, as an example. Let *Y* be the total number of genes in the yeast genome, *S* be the total number of yeast heat shock responsive genes in the yeast genome identified in Step 1, *R* be the total number of Hsf1’s regulatory targets in the yeast genome retrieved from the TF-gene association data set, and *m* be the number of Hsf1’s regulatory targets that respond to heat shock. Then the *p*-value for rejecting the null hypothesis (H_0_: Hsf1 is not involved in responding to heat shock) is calculated as

$$p=P(x\geq m)=\sum_{x\geq m}\frac{\left(\begin{array}{c} S\\ x \end{array}\right)\left(\begin{array}{c} Y-S\\ R-x \end{array}\right)}{\left(\begin{array}{c} Y\\ R \end{array}\right)}.$$

This *p*-value is then adjusted by Bonferroni correction to represent the true alpha level in the multiple hypothesis testing. Hsf1 is said to be involved in responding to heat shock if the adjusted *p*-value *p_adjusted_* ≤ *p_threshold_*.

**Figure 1.**
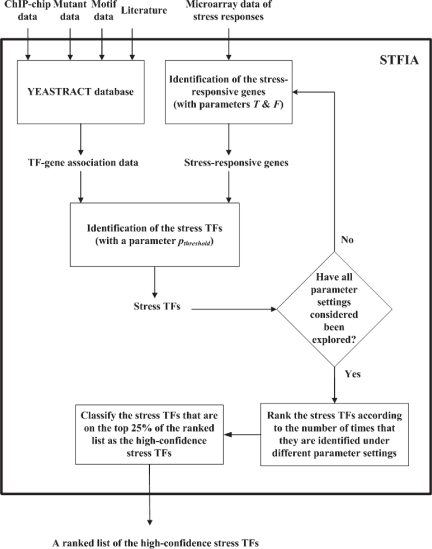
The flowchart of STFIA.

**Step 3:** (exploration of different parameter settings) Repeat Step 1 and Step 2 for different parameter settings of *T*, *F* and *p_threshold_*. In this study, 16 × *L* (*T* = 1, 2,*…* ,*L; F* = 2, 3, 4, 5; *p_threshold_* = 10^−2^, 10^−3^, 10^−4^, 10^−5^) possible parameter settings are investigated. *L* is the length of the gene expression time profile measured under a specific stress. After exploring all these parameter settings, STFIA ranked the stress TFs according to the number of times that they are identified under different parameter settings. That is, the first TF in the list is the one that is identified with the largest number of times. The TFs that are on the top 25% of the ranked list are classified as the high-confidence stress TFs. Finally, STFIA outputs a ranked list of the high-confidence stress TFs for a specific stress. The flowchart of STFIA could be seen in Figure 1.

## Results

### Biologically significant stress TFs have been found

We applied STFIA to find the stress TFs that are involved in responding to heat shock, oxidative shock, osmotic shock, acidic stress, nitrogen depletion, and amino acid starvation, respectively. Table 1 shows the high-confidence stress TFs for each of the above six stresses. The identified stress TFs can be divided into two categories. The first category is the well-known stress TFs with solid literature evidences which could directly indicate involvement of these TFs in response to that specific stress. The second category is the novel stress TFs that have partial (indirect) or no literature supports. We found that 51% (24/47 counting multiplicity) of the predicted stress TFs belongs to the first category, validating the effectiveness of STFIA. Besides, 65% (15/23 counting multiplicity) of the second category has partial (indirect) literature supports, showing the prediction power of STFIA. Therefore, the eight novel stress TFs that have no literature evidences yet are worthy to be experimentally verified in the future.

### Biological validation of our results

Now we discuss in details the evidences from the literature that support our findings.

#### Heat shock

As shown in Table 1, Msn2, Msn4 and Hsf1 are well-known heat shock TFs. Msn2 and Msn4 bind DNA at stress response element (STRE) and activate many STRE-regulated genes in response to many stresses such as heat shock, oxidative shock, osmotic shock, etc (Cherry et al. 1998). Hsf1 binds DNA at heat shock element (HSE) and activates multiple genes in response to hyperthermia (Cherry et al. 1998).

Sfp1, Pdr3, Rpn4 and Stp1 are novel heat shock TFs. Two previous studies partially support our findings. First, Sfp1 regulates RP gene expression in response to heat shock. In response to heat shock, Sfp1 is released from RP gene promoters and leaves the nucleus, and RP gene transcription is down-regulated (Marion et al. 2004). Second, Hsf1 activates expression of *PDR3*, encoding a multidrug resistance TF. Moreover, Hsf1 and Pdr3 both can directly activate expression of *RPN4*, encoding a TF that directly activates expression of a number of genes encoding proteasome subunits. It is demonstrated that the Hsf1 binding site (HSE) in the *RPN4* promoter is primarily responsible for heat induction of *RPN4*, with a minor contribution of Pdr3 binding sites (PDREs). The overlapping transcriptional regulatory networks involving Hsf1 and Pdr3 in regulating the expression of *RPN4* suggest a close linkage between heat shock tolerance and pleiotropic drug resistance (Hahn et al. 2006).

#### Oxidative shock

As shown in Table 1, Msn2, Msn4, Yap1 and Skn7 are well-known oxidative shock TFs. Msn2 and Msn4 activate many STRE-regulated genes in response to many stresses such as oxidative shock, heat shock, osmotic shock, etc (Cherry et al. 1998). Yap1 and Skn7 are known to regulate genes that respond to oxidative shock. For example, they co-operate to regulate *TRX2*, a cytoplasmic thioredoxin isoenzyme of the thioredoxin system which protects cells against oxidative stress (Güldener et al. 2005).

Hsf1 and Pdr3 are novel oxidative shock TFs. The phosphorylation state of Hsf1 changes during oxidative stress, indicating the activity of Hsf1 is modified under oxidative stress (Güldener et al. 2005).

#### Osmotic shock

As shown in Table 1, Hot1, Msn2 and Msn4 are well-known osmotic shock TFs. Hot1 is required for the transient induction of glycerol biosynthetic genes *GPD1* and *GPP2* in response to high osmolarity (Rep et al. 1999; Güldener et al. 2005). The msn2msn4 double deletion mutants exhibit higher sensitivity to severe osmotic stress, indicating Msn2 and Msn4 are involved in responding to osmotic stress (Cherry et al. 1998).

Pdr3, Hsf1, Ino2 and Ino4 are novel osmotic shock TFs. Two previous studies partially support our findings. First, DNA microarray analysis of Pdr3 mutants indicates novel Pdr3 regulatory targets including those induced by NaCl and/or those conferring salt sensitivity upon deletion. These results reveal an unexpected role for Pdr3 in salt tolerance (Onda et al. 2004). Second, it has been shown that Hsf1 is rapidly activated by either hyper- or hypo-osmotic stress, indicating that Hsf1 may serve some physiological functions during osmotic stress (Caruccio et al. 1997).

#### Acidic stress

As shown in Table 1, Msn2 and Msn4 are well-known acidic stress TFs. *RGD1* is known to be activated at low pH and after heat and oxidative shocks. The transcription level at low pH and after heat shock was demonstrated to depend on the STRE box located in the *RGD1* promoter. The general stress-activated transcription factors Msn2 and Msn4 were shown to mainly act on the basal *RGD1* transcriptional level in normal and stress conditions (Gatti et al. 2005).

Pdr3, Stp1, Ino2 and Ino4 are novel acidic stress TFs. Teixeira et al. (2006a) analyzed the global gene transcription pattern of the yeast in response to sudden aggression with the 2,4-dichlorophenoxyacetic (2,4-D) acid. They found that most of the up-regulated genes in response to 2,4-D acid are known targets of Msn2, Msn4, Pdr3 and Stp1, partially validating our findings.

#### Nitrogen depletion

As shown in Table 1, Gln3, Dal80, Dal81 and Gcn4 are well-known nitrogen depletion TFs. Gln3 and Dal80 are known to regulate the expression of nitrogen catabolite pathways in *Saccharomyces cerevisiae*. They bind to motifs in the promoter region to the consensus sequence 5′GATAA3′. Gln3 acts positively on gene expression whereas Dal80 acts negatively (Hofman-Bang, 1999). Dal81 is shown to be required for induced expression of two differently regulated nitrogen catabolic genes (Bricmont et al. 1991). It is shown that nitrogen starvation regulates translation of Gcn4 by a novel mechanism that involves the four upstream open reading frames in the Gcn4 mRNA leader sequence (Grundmann et al. 2001).

Stp1, Stp2, Rpn4, Sfp1, Ifh1, Arr1 and Ino4 are novel nitrogen depletion TFs. Several previous studies partially support our findings. First, stp1stp2 double deletion mutants exhibit a number of transcriptional phenotypes, such as the increased expression of genes subject to nitrogen catabolite repression and genes involved in stress response, indicating that Stp1 and Stp2 play roles in pathways for the assimilation of nitrogen (Eckert-Boulet et al. 2004). Second, on solid growth media with limiting nitrogen source, diploid budding-yeast cells differentiate from the yeast form to a filamentous, adhesive, and invasive form. Both low availability of nitrogen and a solid growth substrate are required to induce diploid filamentous-form growth. It is known that Rpn4 regulates filamentous growth, indicating that Rpn4 may be involved in responding to nitrogen depletion (Prinz et al. 2004). Third, ribosomal protein (RP) genes in eukaryotes are coordinately regulated in response to growth stimuli and environmental stress, thereby permitting cells to adjust ribosome number and overall protein synthetic capacity to physiological conditions. Sfp1 and Ifh1 are known to regulates RP gene expression in response to nutrients depletion (Cherry et al. 1998; Marion et al. 2004).

#### Amino acid starvation

As shown in Table 1, Gcn4, Dal80, Gln3, Met4, Met28, Met31, Leu3 and Gat1 are well-known amino acid starvation TFs. Several previous studies support our findings. First, Gcn4 is a well-known transcriptional activator of amino acid biosynthetic genes in response to amino acid starvation (Cherry et al. 1998). Second, the expressions of the genes encoding the general amino acid permease and the ammonium permease are regulated by Gln3 and Dal80. Another group of genes whose expressions are also regulated by Gln3 and Dal80 are some proteases, *CPS1*, *PRB1*, *LAP1*, and *PEP4*, responsible for the degradation of proteins into amino acids thereby providing a nitrogen source to the cell (Hofman-Bang, 1999). This indicates nitrogen depletion and amino acid starvation responses have regulatory cross-talk because they both use Gln3 and Dal80 to regulate genes in response to both stresses. Third, Met4, Met28 and Met31 are lecine-zipper transcriptional activators, responsible for the regulation of the sulfur amino acid pathway (Cherry et al. 1998). Fourth, Leu3 is known to activate multiple genes for branched-chain amino acid biosynthesis (Friden and Schimmel, 1988). Fifth, Gat1 is known to regulate the general amino acid permease gene *GAP1*, the glutamine synthetase gene *GLN1*, and *CAR1*, *ASP3*, *PUT1*, and *PUT2*, which encode enzymes involved in the degradation of arginine, asparagine, and proline respectively (Cherry et al. 1998).

Stp1 and Stp2 are novel amino acid starvation TFs. One study partially supports our prediction (Ecker-Boulet et al. 2004). *S. cerevisiae* responds to the presence of amino acids in the environment through the membrane-bound complex SPS, by altering transcription of several genes. Global transcription analysis shows that 46 genes are induced by L-citrulline. Under the given conditions there appears to be only one pathway for induction with L-citrulline, and this pathway is completely dependent on the SPS component, Ssy1, and either of the transcription factors, Stp1 and Stp2 (Ecker-Boulet et al. 2004).

## Discussion

To have the ability to respond rapidly to abrupt and dramatic fluctuation in the external conditions is crucial for cell survival. Sudden changes in the external environment can perturb the internal system of the cells, disrupting cellular functions and preventing growth. Therefore, unicellular organisms such as yeasts have evolved complex adaptation mechanisms to cope with environmental stresses. One aspect of this cellular adaptation is the reorganization of genomic expression (Gasch and Werner-Washburne, 2002). The genome-wide stress-responsive genes in yeast have been discovered by DNA microarrays (Gasch et al. 2000; Causton et al. 2001). However, the complete network of the regulators of stress responses and the details of their actions remain to be elucidated (Gasch et al. 2000).

In this study, we developed STFIA which integrates gene expression and TF-gene association data to identify stress TFs of six kinds of stresses. Some general stress TFs that are in response to various stresses and some specific stress TFs that are in response to one specific stress are identified. For example, STFIA found out the general stress TFs Msn2 and Msn4 (Cherry et al. 1998) and the well-known heat shock TF Hsf1 (Cherry et al. 1998), oxidative shock TFs Skn7 and Yap1 (Güldener et al. 2005), osmotic shock TF Hot1 (Rep et al. 1999), nitrogen depletion TFs Gln3 and Dal80 (Hofman-Bang, 1999), and amino acid starvation TFs Gcn4, Met4, Met28 and Met31 (Cherry et al. 1998). The ability to find out these well-known stress TFs validates the power of STFIA.

**Figure 2.**
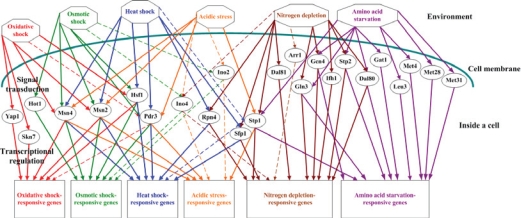
The transcriptional regulatory network of the yeast stress response. Environmental stresses are represented by octagons, stress TFs are represented by ellipses, and stress-responsive genes are represented by rectangles. Solid (Dashed) lines indicate the known (predicted) regulatory relationships among environmental stresses, stress TFs, and stress-responsive genes. Oxidative shock responses are colored red, osmotic shock responses are colored green, heat shock responses are colored blue, acidic stress responses are colored orange, nitrogen depletion responses are colored brown, and amino acid starvation responses are colored purple.

**Figure 3.**
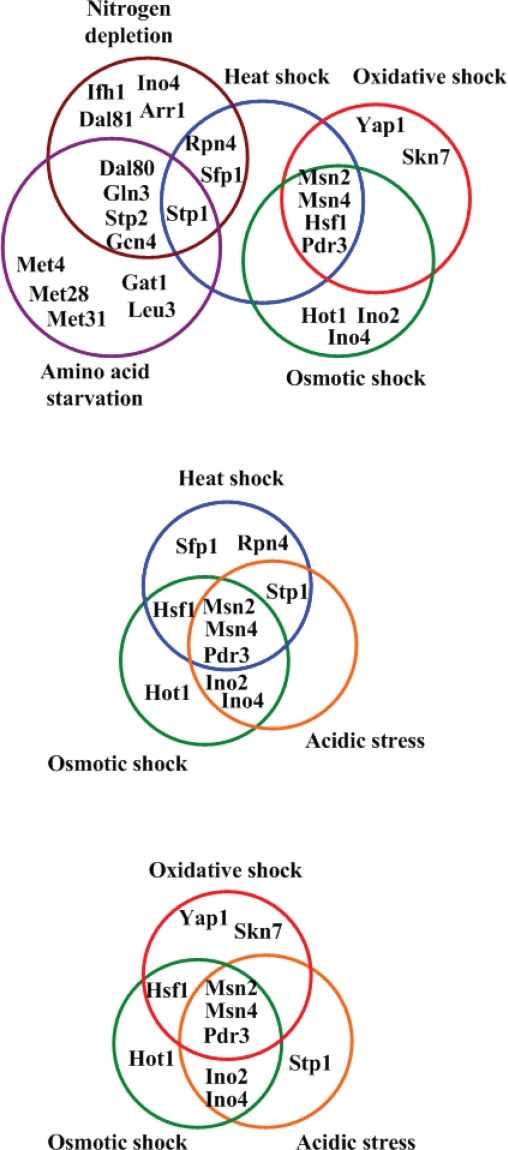
Regulatory cross-talk among different stress responses. The cellular responses to heat shock, oxidative shock, osmotic shock and acidic stress have significant regulatory cross-talk. They all trigger stress TFs Msn2, Msn4 and Pdr3. Besides, nitrogen depletion and amino acid starvation responses have regulatory cross-talk. They both trigger stress TFs Gcn4, Gln3, Dal80, Stp1 and Stp2.

STFIA identified 24 distinct TFs (Arr1, Dal80, Dal81, Gat1, Gcn4, Gln3, Hot1, Hsf1, Ifh1, Ino2, Ino4, Leu3, Met28, Met31, Met4, Msn2, Msn4, Pdr3, Rpn4, Sfp1, Skn7, Stp1, Stp2, and Yap1) to be in response to at least one of the six stresses under study (see Fig. 2). That is, a small number of TFs may be sufficient to control a wide variety of expression patterns in yeast under different stresses. Two implications can be inferred from this observation. First, the adaptation mechanisms to different stresses may have a bow-tie structure (Csete and Doyle, 2004). As shown in Figure 2, the core stress TFs make up the ‘knots’ of a bow tie, facilitating the fan in of a large of variety of environmental stresses through signal transduction pathways and fan out of an even larger variety of stress-adapting proteins through activating stress-responsive target genes. Actually, approximately two-thirds of the yeast genome (about 4000 genes) is involved in responding to the changes in environment (Causton et al. 2001). Second, there exists extensive regulatory cross-talk between different stress responses (see Fig. 3). We found that heat shock, oxidative shock, osmotic shock, and acidic stress all can trigger the stress TFs Msn2, Msn4 and Pdr3, indicating these four stresses share a similar stress adaptation mechanism. Moreover, we found that nitrogen depletion and amino acid starvation both can trigger the stress TFs Gcn4, Gln3, Dal80, Stp1 and Stp2, indicating a cross-talk between the cellular responses to these two stresses. This is not surprising because both nitrogen depletion and amino acid starvation belong to the nutrient deprivation stress and could have a similar stress adaptation mechanism. The fact that different stress adaptation mechanisms share some, but not all, of their regulators suggests a higher level of modularity of the yeast stress response network (Segal et al. 2003).

**Figure 4.**
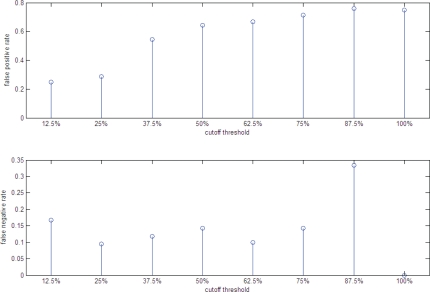
Statistics of the performance of STFIA using different cutoff threshold. The false positive and false negative rates of STFIA using different cutoff threshold are shown. When the cutoff threshold equals 25%, STFIA has the best performance in terms of the trade-off between the false positive and false negative rates to find out the high-confidence heat shock TFs.

Step 3 of STFIA is very crucial for filtering out the low-confidence stress TFs that may be found only under some specific parameter setting. For example, 15 TFs (Arr1, Fhl1, Gcn4, Hsf1, Ifh1, Msn2, Msn4, Pdr3, Rap1, Rgt1, Rpn4, Rtg1, Rtg3, Sfp1, Stp1) are identified in Step 2 of STFIA as the heat shock TFs with the parameter setting *T* = 1, *F* = 2, and *p_threshold_* = 0.01. However, nine (Arr1, Fhl1, Gcn4, Ifh1, Rap1, Rgt1, Rtg1, Rtg3, Stp1) of these 15 TFs are with low confidence because no known evidence shows that they are involved in responding to heat shock. As shown in Table 1, eight of the nine low-confidence stress TFs are eliminated in Step 3 of STFIA because they are not on the top 25% of the ranked list. The reason for choosing only the top 25% of the ranked list is as follows. If we choose a more stringent cutoff threshold, we will not have enough stress TFs (say five) for each stress to see whether there exists cross-talk among different stresses. However, if we choose a looser cutoff threshold, we may include some low-confidence stress TFs into our results. In Figure 4, we show that when the cutoff threshold equals 25%, STFIA has the best performance in terms of the trade-off between the false positive and false negative rates to find out the high-confidence heat shock TFs.

## Conclusion

The adaptation mechanisms to stress are highly complex. They require a complex network of sensing and signal transduction leading to adaptations of cell growth and proliferation as well as to adjustments of the gene expression program, metabolic activities, and other features of the cell (Hohmann and Mager, 2003). This study focuses on the regulation of the genomic expression, which is only one part of cellular adaptation mechanisms. Nevertheless, our study proposed a network of the regulators of stress responses and their mechanism of action. Thus, it provides a starting point for understanding the mechanisms that yeast uses to survive some of the environmental conditions that cells experience in the wild. We believe that as more genomic expression data emerge, in combination with data from other whole-organism approaches, novel computational algorithms such as STFIA have potential to construct a dynamic picture of the integrated cellular response of yeast cells to environmental changes.

## Figures and Tables

**Table 1.** The high-confidence stress TFs involved in responding to each of the six stresses. The high-confidence stress TFs involved in responding to heat shock, oxidative shock, osmotic shock, acidic stress, nitrogen depletion and amino acid starvation are shown. The TFs for each stress are ranked according to the number of times that they are identified under different parameter settings. For example, among all osmotic shock TFs, Hot1 is the one that is identified with the largest number of times. Besides, the stress TFs are colored blue (red) if there exist solid (partial) literature evidences showing that they are involved in responding to the same stress as we predicted.

**Heat shock TFs**	**Oxidative shock TFs**	**Osmotic shock TFs**	**Acidic stress TFs**	**Nitrogen depletion TFs**	**Amino acid starvation TFs**
Msn4	Msn4	Hot1	Msn4	Gln3	Gcn4
Msn2	Msn2	Msn4	Msn2	Dal80	Dal80
Hsf1	Yap1	Msn2	Pdr3	Stp1	Met28
Pdr3	Skn7	Pdr3	Ino2	Stp2	Gln3
Stp1	Pdr3	Hsf1	Ino4	Sfp1	Met4
Sfp1	Hsf1	Ino2	Stp1	Arr1	Stp1
Rpn4		Ino4		Rpn4	Stp2
				Dal81	Leu3
				Gcn4	Gat1
				Ifh1	Met31
				Ino4	
